# ‘It gives a sense of purpose’: a survey exploring GP registrars’ views on relational continuity of care

**DOI:** 10.3399/BJGPO.2025.0001

**Published:** 2025-09-24

**Authors:** Helen Leach, Helen Atherton, Jeremy Dale

**Affiliations:** 1 Warwick Medical School, University of Warwick, Coventry, UK; 2 Primary Care Research Centre, University of Southampton, Southampton, UK

**Keywords:** continuity of patient care, postgraduate education, statistics, general practice

## Abstract

**Background:**

Relational continuity of care has been shown to improve patient outcomes and clinician satisfaction. However, little is known about how GP registrars, the future workforce in general practice, experience this continuity, especially in the context of evolving workforces and increasing use of remote consultations.

**Aim:**

To explore GP registrars’ views on relational continuity of care and identify personal, training, and practice factors that may influence these views, including the impact of digital or remote consulting.

**Design & setting:**

A cross-sectional online survey was conducted among GP registrars in the West Midlands, England. The survey explored registrars’ experiences and attitudes towards relational continuity, including the impact of remote consulting.

**Method:**

The survey combined Likert-scale and multiple-choice questions with free-text responses. Quantitative data were analysed using descriptive statistics with logistic regression to identify personal and training factors that might influence views. Content analysis was used for qualitative data.

**Results:**

In total, 246 responses were received (estimated 14.5% response rate). Registrars valued relational continuity, particularly for patients with long-term conditions. Barriers such as rotational training, high workload, and limited appointment access were reported. Remote consulting was seen as challenging for building relationships but helpful for follow-up encounters. Few personal or training factors influenced views on relational continuity.

**Conclusion:**

Despite significant barriers, GP registrars highlighted the importance of relational continuity for patient care and clinician satisfaction. Further research is needed to explore how training models impact continuity, and how training and remote consulting can support the experience of relational continuity in practice.

## How this fits in

Relational continuity of care is valued for improving patient outcomes and clinician satisfaction, but little is known about how GP registrars experience it. Previous research has mainly focused on fully qualified GPs, with limited attention to registrars’ perspectives. This study finds that GP registrars highly value relational continuity but face significant barriers such as rotational training and high workloads. This research highlights the need for training models that support relational continuity of care and a greater need to understand the impact of remote consulting.

## Introduction

Relational continuity of care is often regarded as a hallmark of general practice and, for many clinicians and patients, is intertwined with ‘relationship-based care’.^1^ Relational continuity refers to the development of a therapeutic relationship with patients over time. There are other forms of continuity; for example, managerial continuity, which refers to coordination of the patient care journey and informational continuity, which involves staff having access to accurate up-to-date patient records.^2^ Evidence has demonstrated that relational continuity of care is valued in general practice internationally.^3,4^

Literature exploring relational continuity has demonstrated reduced hospitalisation and mortality,^5–7^ safer prescribing,^8^ and improved job satisfaction.^9^ Patients also value relational continuity, increasing trust and improving clinical care.^10,11^ The UK Government has highlighted the importance of relational continuity,^12^ with suggestions at a policy level that general practice should include and support this continuity.^2,13^

However, little research has explored how registrars, as future independent clinicians, experience relational continuity. GP registrars are an increasingly heterogeneous group from a diverse range of personal and clinical backgrounds with varied experiences and expectations of general practice.^14^ While relational continuity of care is mentioned throughout the UK training curriculum documents, including specific reference to ‘*maintain a continuing relationship with patients, carers and families’*,^15^ experience of continuity within training is modest, with lower patient satisfaction with continuity at training practices.^16,17^ This may be related to rotational training, patient preference to see a ‘usual’ GP, and/or a reconceptualisation of continuity to being multidisciplinary team-based as opposed to between individual clinician and patient.^16,18^

Today’s registrars are working in an environment with more use of remote consultation than ever, with >30% of patient contacts occurring remotely in 2024 in England.^19^ Since 2020, they have undertaken final examinations that have included telephone and video consulting with patients.^20,21^ They are affected by the lack of high-quality educational literature on how to train in digital and remote consulting,^22,23^ and a lack of understanding of the impact of remote consulting on relational continuity.^24^

Given the sparsity of literature examining GP registrars’ views on relational continuity, this work aimed to explore those views and what personal, training, and practice factors, including the use of remote consulting, may influence them.

## Method

### Survey development

The survey was developed by the research team, which included two GPs and a researcher experienced in survey methodology (see Supplementary Appendix S1).

The survey collected personal characteristics of participants, including age, sex and ethnicity, and educational backgrounds, such as stage of training, Membership of Royal College of General Practitioners (MRCGP) examinations undertaken, and previous specialty training. These questions were used to identify potential factors that could influence views towards relational continuity of care. We defined relational continuity in the survey at the beginning of the questions about continuity as *‘the ongoing therapeutic relationship between a patient and one or more providers’*.

Experiences and views of remote or digital consulting were explored and these questions drew on previous surveys by the research team.^25,26^

Views and experiences of continuity were explored through a mixture of short-answer, multiple-choice, and Likert-scale questions.

The survey was piloted and collaboratively modified with four GPs who had recently completed training, each drawing on their recent experience as GP registrars. This ensured that the pilot group would not overlap with potential participants but would include input from those with recent experience as registrars.

### Recruitment

GP registrars working in the West Midlands were eligible for inclusion in this survey. When the survey was developed, approximately 1700 GP registrars were based in the West Midlands (Table 1). Recruitment materials and the study information sheet stated that the survey was for registrars in the West Midlands.

**Table 1.** GP training in the UK, with reference to the West Midlands

Training lasts 3 years full-time (ST1–3). It is elongated as required for equivalent part-time. A fourth year (ST4) can be undertaken for a fellowship opportunity
There is a national curriculum and overall training pathway Posts and programmes are arranged by local training teams, known as vocational training schemes Any qualified medical practitioner can apply for GP training and registrars may have varied experience before joining the GP training scheme The first 18 months is spent on rotations in hospitals 18–24 months is then spent within general practice Registrars do 3–6 month rotational placements during training, with the final 12 months spent within one general practice Some regions offer longitudinal placements where time may be spent earlier in training in the same practice before returning for the final year In the West Midlands, there are five vocational training schemes: Birmingham and Solihull, Sandwell and Black Country, Coventry and Warwickshire, Hereford and Worcestershire, and Staffordshire and Shropshire At the time of the initial survey analysis, there were 1716 doctors enrolled in general practice training in the West Midlands. In total,1202 were undertaking a placement within general practice itself Across the region, there were 517 (30.1%) ST1s, 520 (30.3%) ST2s, and 679 (39.6%) ST3/4s (one coded as unknown)

ST = specialty training.

The survey was hosted online by Qualtrics between July and November 2024. It was disseminated by email and via social media by the Workforce, Training and Education team (NHS England Midlands team) and the Royal College of General Practitioners Midland Faculty. There was also distribution through local vocational training schemes across the West Midlands where training programme directors supported advertisement at scheduled teaching sessions.

### Data analysis

Statistical data were analysed in SPSS (version 29). The first stage was a descriptive analysis of personal and training characteristics. The second stage used a logistic regression model that explored the link between personal and training characteristics with views regarding continuity and the use of remote consulting. Free-text answers were analysed using inductive content analysis, which involved compiling the free-text answers into a dataset, reading them, and organising them into themes and reporting themes alongside relevant examples from the data.^27^

## Results

In total, 246 survey responses were received, giving a response rate of approximately 14.5%. Owing to the fluctuating nature of training, with registrars entering and leaving training throughout the year, it was not possible to calculate an exact response rate.

Responders’ characteristics are represented in Table 2. Most were female, participants were a range of ages, and there was ethnic diversity in the sample. When compared with available data on the full population of registrars, which was obtained directly from NHS England via a freedom of information request, the responders were aligned with the wider population of registrars in relation to sex (61.8% female in sample, 60.4% in wider population) and place of medical education (61.0% international medical graduates [IMGs] in sample, 62.4% in wider population). The overall number of registrars in ethnic minority groups was 75.6% in the sample and 79.1% in the wider population. See Supplementary Table S1 for all available comparator information.

**Table 2.** Personal and training characteristics

Characteristic	Category	*n*	%
**Age, years**	25–29	45	18.3
	30–34	98	39.8
	35–39	58	23.6
	40–44	35	14.2
	Other	8	3.3
	Prefer not to say	2	0.8
**Sex**	Female	152	61.8
	Male	91	37.0
	Prefer not to say	3	1.2
**Ethnicity**	Asian or Asian British	100	40.7
	Black, Black British, Caribbean or African	64	26.0
	White	53	21.5
	Middle Eastern	12	4.9
	Mixed or multiple ethnic groups	7	2.8
	Other	3	1.2
	Prefer not to say	7	2.8
**Carer status^a^**	No response provided	1	0.4
	None	109	44.3
	Prefer not to say	10	4.1
	Primary carer of a child or children	118	48.0
	Primary carer of an adult	4	1.6
	Primary carer of an older person	5	2.0
	Secondary carer	6	2.4
**Presence of disability or long-term condition**	No	208	84.6
	Yes	29	11.8
	Prefer not to say	9	3.7
**Training year**	ST1	85	34.6
	ST2	64	26.0
	ST3+	78	31.7
	No response provided	19	7.7
**Currently in GP post**	No	64	26.0
	Yes	166	67.5
	No response provided	16	6.5
**MRCGP examinations passed**	Applied Knowledge Test only	58	23.6
	Applied Knowledge Test and Recorded Consultation Assessment or Simulated Consultation Assessment	26	10.6
	No examinations passed	153	62.2
**Place of medical education**	European Economic Area graduate	8	3.3
	International medical graduate	150	61.0
	UK graduate	86	35.0
	No response provided	2	0.8
**Previous ST**	No	187	76.0
	Yes	57	23.2
	No response provided	2	0.8

^a^Frequencies add up to >246 owing to seven responders selecting >1 option. MRCGP = Membership of the Royal College of General Practitioners. ST = specialty training.

More than half of the responders identified caring responsibilities. A small proportion lived with a disability or long-term health condition (Table 2).

There were responses from registrars across all training years. Most had not passed any MRCGP examinations, possibly reflecting the proportion of ST1 responders (Table 2). Nearly one-quarter of responders had trained in another specialty before GP training (see Supplementary Table S2).

### Benefits of relational continuity of care

Registrars responded positively to the benefits to their clinical practice from relational continuity (Table 3), with 74.8% (*n* = 184) responding that it was ‘very’ or ‘extremely’ important for them to build a relationship with a patient. Around three-quarters also felt that relational continuity was ’very’ or ‘extremely’ important for benefitting the health of their patients (74.4%, *n* = 183) and for efficiency (72.8%*, n* = 179). Job satisfaction and finding out clinical outcomes were more moderately regarded as important. Free-text comments can be found in Supplementary Table S3.

**Table 3.** What are the benefits of relational continuity for care (being part of) your clinical practice?

Statement	Response	*n*	%
It is important for me to build a relationship with a patient	Extremely important	91	37.0
	Very important	93	37.8
	Moderately important	18	7.3
	Slightly important	2	0.8
	Not sure	1	0.4
	No response provided	41	16.7
My consultations are more efficient when I know the patient	Extremely important	108	43.9
	Very important	71	28.9
	Moderately important	19	7.7
	Slightly important	2	0.8
	Not at all important	2	0.8
	Not sure	3	1.2
	No response provided	41	16.7
I find out outcomes from my clinical decision making, for example, prescribing	Extremely important	79	32.1
	Very important	85	34.6
	Moderately important	36	14.6
	Slightly important	1	0.4
	Not at all important	1	0.4
	Not sure	3	1.2
	No response provided	41	16.7
It improves my job satisfaction	Extremely important	97	39.4
	Very important	72	29.3
	Moderately important	30	12.2
	Slightly important	5	2.0
	Not at all important	1	0.4
	No response provided	41	16.7
It benefits the health of my patients	Extremely important	104	42.3
	Very important	79	32.1
	Moderately important	18	7.3
	Slightly important	3	1.2
	Not sure	1	0.4
	No response provided	41	16.7

Those who had not undertaken specialty training before embarking on GP training were more likely to report that relational continuity was ‘very’ or ‘extremely’ important for job satisfaction (odds ratio [OR] 2.75, 95% confidence interval [CI] = 1.18 to 6.39, *P* = 0.019) (see Supplementary Table S4). IMG and European Economic Area (EEA) graduates (non-UK graduates combined) were less likely to report that benefits of relational continuity were ‘very’ or ‘extremely’ important for benefitting the health of patients (OR 0.077, 95% CI = 0.01 to 0.62, *P* = 0.016) or for efficiency in consulting (OR 0.07, 95% CI = 0.01 to 0.39, *P* = 0.002) (see Supplementary Tables S5 and S6). There were no other statistically significant relationships identified between any further personal or training characteristics and the benefits of relational continuity. See Supplementary Appendix S1 for more details.

### The importance of relational continuity of care

Registrars were asked about the importance of relational continuity for varying patient and staff groups (see Supplementary Table S7). While 169 participants selected that it was ‘very’ or ‘extremely’ important for all patients, this did not extend to some patient groups and presentations. Relational continuity was felt to be most important for patients with long-term conditions (78.9%, *n* = 194), mental health conditions (76.8%, *n* = 189), intellectual disability (71.5%, *n* = 176), or of older age (78.0%, *n* = 192). The two patient groups where relational continuity was felt least important were those with acute presentations (for example, a viral respiratory tract infection) or those aged <18 years (see Supplementary Tables S8 and S9).

Relational continuity was felt to be particularly important for GPs and GP registrars to deliver, with 71.1% (*n* = 175) and 63.4% (*n* = 156) selecting ‘very’ or ‘extremely’ important (see Supplementary Table S10). No responders selected ‘not important’ for either group. There were more mixed views for other clinical team members, particularly for advanced care practitioners (ACPs), medical associate professions (MAPs; for example, physician associates) and healthcare assistants (HCAs); MAPs and HCAs were more frequently rated as ‘slightly’ or ‘not at all’ important (16.3%, *n* = 40 and 19.1%*, n* = 47, respectively).

### Barriers to relational continuity of care

Around half of the registrars responded that they ‘definitely’ or ‘probably’ were delivering relational continuity in their practice (54.0%, *n* = 133), with 17.0% (*n* = 42) responding that they were ‘definitely not’ or ‘probably not’ delivering it (data not shown).

In total, 215 registrars identified ≥2 barriers to delivering relational continuity (Table 4). Rotational training was the biggest barrier (46.7%, *n* = 115). Ninety-one responders (37.0%) selected ‘overwhelming workload’ as a barrier, with around one-quarter (23.6%, *n* = 58) identifying training requirements such as portfolio completion or revision as a barrier. Further barriers included access to appointments for patients and access to appointments that registrars could book for their patients.

**Table 4.** Barriers to relational continuity of care

Barrier	*n* ^a^	%
Do not know enough about it	4	1.6
Less than full-time working	45	18.3
Not a priority for clinical practice	30	12.2
Overwhelming workload	91	37.0
Patients find it difficult to book appointments	98	39.8
Rotational training	115	46.7
There are no barriers	6	2.4
Training requirements, for example, portfolio or revision commitments	58	23.6
Unable to book own patients into appointments	94	38.2
Other	11	4.5
No response provided	43	17.5

^a^≥2 barriers were identified by 215 registrars so frequencies add up to >246.

’Other’ barriers included a focus on urgent access (at local and policy level), with a lack of pre-bookable appointments, the use of ACPs or MAPs for acute presentations, and 10-minute appointments. Further concerns about workload, particularly administrative burden, were detailed.

### The impact of digital and remote consulting

Most registrars were currently undertaking face-to-face consultations with patients (74.4%*, n* = 183); however, many had experience with telephone (75.6%, *n* = 186) and online (35.0%, *n* = 86) consulting. Very few registrars had undertaken video consultations (13.0%, *n* = 32), and a small proportion had only ever undertaken face-to-face consulting (4.9%, *n* = 12) (see Supplementary Tables S11 and S12).

Overall, 43.1% (*n* = 106) felt that it was more difficult to build relationships with patients via a remote consultation (‘somewhat’ or ‘strongly’ disagree), but consulting was felt to be easier in the context of a pre-existing relationship (Table 5). There were mixed views about whether remote consulting made relational continuity easier, although nearly half of the responders stated that they would be more likely to follow a patient up if they could do so remotely (44.7%*, n* = 110). There were no significant relationships between personal or training characteristics and views on continuity in remote consulting (see Supplementary Table S13). There was also no significant relationship between views and use of consulting modalities.

**Table 5.** Responses to the question, *‘In your experience, how does remote consulting impact on relational continuity of care in general practice?’*

Statement	Response	*n*	%
Remote consulting makes it easy for me to provide continuity of care	Strongly agree	15	6.1
	Somewhat agree	89	36.2
	Neither agree nor disagree	58	23.6
	Somewhat disagree	28	11.4
	Strongly disagree	12	4.9
	No response provided	44	17.9
It is easy to consult remotely with patients I do not know	Strongly agree	2	0.8
	Somewhat agree	28	11.4
	Neither agree nor disagree	53	21.5
	Somewhat disagree	76	30.9
	Strongly disagree	42	17.1
	No response provided	45	18.3
It is easy to consult remotely with patients I do know	Strongly agree	46	18.7
	Somewhat agree	108	43.9
	Neither agree nor disagree	35	14.2
	Somewhat disagree	10	4.1
	Strongly disagree	2	0.8
	No response provided	45	18.3
I find it easy to build relationships with patients via a remote consultation	Strongly agree	2	0.8
	Somewhat agree	32	13.0
	Neither agree nor disagree	62	25.2
	Somewhat disagree	78	31.7
	Strongly disagree	28	11.4
	No response provided	44	17.9
I am more likely to follow a patient up if I can do it remotely	Strongly agree	33	13.4
	Somewhat agree	77	31.3
	Neither agree nor disagree	59	24.0
	Somewhat disagree	25	10.2
	Strongly disagree	8	3.3
	*Blank*	44	17.9

### Understanding relational continuity of care

In total, 189 registrars described what relational continuity of care meant for them in free-text answers (see Supplementary Table S14). The key themes are summarised in Table 6. The majority conceptualised relational continuity of care as building individual longitudinal relationships with patients, to develop rapport and trust, and to deliver personalised care inclusive of biopsychosocial factors. Forty-two responders considered continuity as aligning with ‘follow-up’ of clinical decisions, typically associated with episodes of care or symptoms. Seven considered continuity as based within a team of clinicians.

**Table 6.** GP registrars’ conceptualisation of relational continuity of care

Category	Examples
Building and maintaining relationships	**Rapport and trust** *‘This personalised approach promotes trust, better communication, and more effective management of long-term health conditions.’* **Holistic, personalised care** *‘It fosters a deeper understanding of patients’ histories and needs, ultimately leading to better health outcomes and patient satisfaction.’* **Between two individuals** *‘The cornerstone of GP- seeing the same GP each time.’* *‘Seeing the same person so they are aware of your ongoing issues.’*
Coordinated clinical care	**Information sharing** *‘This means that patient care is joined up, documentation helps transfer seamless care of condition(s) for a patient.’* **Follow-up** *‘Following a patient up after you have seen them and started an intervention.’* **Team-based care** *‘Coordination between a clinician/clinical team and patient in the provision of their ongoing care.’* *‘The seemless* [sp] *transition of care horizontally between primary care clinicians and vertically with secondary care providers.’*
Improved satisfaction in care	**Better job satisfaction** *‘Continuity of care is the whole point of general practice in my opinion.’* *‘It gives a sense of purpose and in a way I’m able to see the good I’m actually doing, in a time when doctors in the UK are feeling underappreciated.’* **Better health outcomes** *‘It is a system* [that] *minimises gaps or oversight in the care of the patient, leading to better quality.’* **Improves patient experience of seeking care** *‘Patients seeing the same doctor again and again to avoid having to start from scratch each time.’*

## Discussion

### Summary

GP registrars valued relational continuity as a core aspect of general practice, recognising its benefits for patients and clinicians, while also noting barriers to its implementation. The majority believed it improved patient health through better clinical outcomes, trust, and communication, particularly for older patients, those with mental health issues, and those with long-term conditions.

Relational continuity was viewed as central to the profession, with one responder stating it was ‘*the whole point of general practice’*. It enhanced job satisfaction and consulting efficiency. These insights suggest continuity is crucial for attracting individuals to general practice.

Registrars identified considerable barriers to relational continuity, including overwhelming workloads owing to urgent access demands and appointment shortages. The biggest barrier was rotational training; other training barriers included the demands of portfolios or exams.

### Strengths and limitations

This novel study showed that GP registrars value relational continuity, highlighting why resident doctors are attracted to general practice training. However, we acknowledge that despite providing a definition of relational continuity of care in the survey, responders may have interpreted continuity differently or may have their own understanding of relational continuity of care.

This study involved newly qualified GPs in the development process. HL, an academic GP, completed her training just before this study, bringing recent experience. Early development included newly qualified GPs, enhancing the validity of the survey to reflect contemporary training experiences and barriers to relational continuity and remote consulting.

The response rate to this survey was approximately 14.5%. The survey was promoted through email, social media, and local training schemes, yet the responders represented only a small fraction of eligible registrars. It was not possible to validate the number of eligible registrars who were aware of the survey, and hence the response rate may have been larger than that estimated. In addition, the survey opened in late summer 2024 after delays, coinciding with potential registrar leave or rotational changes, along with registrars possibly facing survey fatigue owing to frequent feedback requests. Conducting this type of survey earlier in the year may better accommodate training schedules and have resulted in a greater level of response.

Despite a low response rate, the responders reflected the demographics of the GP registrar population in the West Midlands in relation to sex, ethnicity, and place of medical education. There were also responses from across all training year groups. This strengthens the results and their generalisability across the wider training cohort. We acknowledge the possibility that registrars outside of the West Midlands may have taken part, and that we cannot validate the identity of responders as GP registrars and this is a limitation.

### Comparison with existing literature

Much of the literature on relational continuity of care in general practice has focused on the views of qualified GPs, often neglecting registrars’ experiences. In an existing study that included registrars, relational continuity was valued and linked to the purpose of being a GP.^3^ This mirrors broader work highlighting holistic care and one-on-one relationships as motivations for entering general practice.^28^ However, concerns exist that registrars lack opportunities for ongoing responsibility for patients, limiting their exposure to relational continuity.^16,29^ A research study in Australia highlighted that registrars had limited exposure to continuity for older patients with long-term conditions.^30^ Further consideration of how to integrate relational and longitudinal continuity within training programmes may enhance not only understanding but also reinforce what may have been a key motivator for becoming a GP, thus improving job satisfaction.

Existing evidence acknowledges that workload pressures hinder the ability to deliver relational continuity,^31,32^ yet no studies, to our knowledge, have assessed how training demands further disrupt it. Requirements such as e-portfolio completion contribute to heightened stress and have been found to detract from essential learning opportunities.^33–35^ GP registrars report significant burnout.^36^ This study’s findings suggest that training programme demands negatively affect engagement with relational continuity, indicating a need to consider how training is delivered to support registrars in developing the skills to deliver and engage with continuity.

The registrars within this survey felt that building a relationship was harder via remote modalities, a concern similarly raised by patients and in the wider literature on delivering personalised care.^37,38^ However, a systematic review highlighted that remote consulting could facilitate the development of a relationship with prompt communication and enhanced trust;^39^ this is similarly reflected within the survey results, with registrars indicating they might be more likely to follow a patient up if able to do so remotely.

### Implications for research and practice

This work highlights the need to consider how best to support GP registrars to engage with relational continuity throughout training, particularly given the importance placed on it. While the time spent in general practice itself during training has increased from 18 months to up to 2 years, short rotations or integrated training posts may impact experiences of relational continuity. This work suggests that enhancing experiences of relational continuity is likely to be important; suggestions may include longitudinal placements or further extending training from 3 years to 5 years. Within training, the requirement to complete a portfolio could be revisited to understand how it might better support relational continuity of care.

Many of the responders received their medical training outside of the UK. These participants were less likely to report a benefit for patients of relational continuity of care. This differential could be considered when delivering training or designing future research. This work also highlights the need to further understand the impact of remote and digital consulting on relational continuity of care.

The continued expansion of digital and remote services within primary care means that future research might collect information about practice set-up in relation to delivery of consultations and prioritisation of continuity.

In conclusion, registrars emphasised the significance of relational continuity. Although barriers such as training demands and workload were reported, along with mixed opinions on the role of digital consulting, the advantages for staff and patients were acknowledged. Concerns about the existence of relational continuity in modern practice have emerged. Nevertheless, based on registrars’ perspectives in this survey, it remains a fundamental aspect of primary care.
